# GWAS-based polygenic risk scoring for predicting cerebral artery dissection in the Chinese population

**DOI:** 10.1186/s12883-024-03759-0

**Published:** 2024-07-25

**Authors:** Shufan Zhang, Dongliang Zhu, Zhengyu Wu, Shilin Yang, Yuanzeng Liu, Xiaocui Kang, Xingdong Chen, Zhu Zhu, Qiang Dong, Chen Suo, Xiang Han

**Affiliations:** 1 Department of Neurology, Huashan Hospital, Fudan University, Shanghai, China; 2 State Key Laboratory of Genetic Engineering, School of life Sciences, Human Phenome Institute, Fudan University, Shanghai, China; 3 Fudan University Taizhou Institute of Health Sciences, Taizhou, Jiangsu China; 4 Department of Geriatrics, Huashan Hospital, Fudan University, Shanghai, China; 5 Gu Mei Community Health Service Center of Minhang District, Shanghai, China; 6 Department of Neurology, Shanghai Shidong Hospital, Shanghai, China; 7 Department of Neurology, Indianan University Health, Bloomington, IN USA

**Keywords:** Cerebral artery dissection, Genetic risk, Polygenic risk score, Predictive model

## Abstract

**Objective:**

Cerebral artery dissection (CeAD) is a rare but serious disease. Genetic risk assessment for CeAD is lacking in Chinese population. We performed genome-wide association study (GWAS) and computed polygenic risk score (PRS) to explore genetic susceptibility factors and prediction model of CeAD based on patients in Huashan Hospital.

**Methods:**

A total of 210 CeAD patients and 280 controls were enrolled from June 2017 to September 2022 in Department of Neurology, Huashan Hospital, Fudan University. We performed GWAS to identify genetic variants associated with CeAD in 140 CeAD patients and 210 control individuals according to a case and control 1:1.5 design rule in the training dataset, while the other 70 patients with CeAD and 70 controls were used as validation. Then Kyoto Encyclopedia of Genes and Genomes (KEGG) pathway and Gene Ontology (GO) enrichment analyses were utilized to identify the significant pathways. We constructed a PRS by capturing all independent GWAS SNPs in the analysis and explored the predictivity of PRS, age, and sex for CeAD.

**Results:**

Through GWAS analysis of the 140 cases and 210 controls in the training dataset, we identified 13 leading SNPs associated with CeAD at a genome-wide significance level of *P* < 5 × 10^− 8^. Among them, 10 SNPs were annotated in or near (in the upstream and downstream regions of ± 500Kb) 10 functional genes. rs34508376 (OR2L13) played a suggestive role in CeAD pathophysiology which was in line with previous observations in aortic aneurysms. The other nine genes were first-time associations in CeAD cases. GO enrichment analyses showed that these 10 genes have known roles in 20 important GO terms clustered into two groups: (1) cellular biological processes (BP); (2) molecular function (MF). We used genome-wide association data to compute PRS including 32 independent SNPs and constructed predictive model for CeAD by using age, sex and PRS as predictors both in training and validation test. The area under curve (AUC) of PRS predictive model for CeAD reached 99% and 95% in the training test and validation test respectively, which were significantly larger than the age and sex models of 83% and 86%.

**Conclusions:**

Our study showed that ten risk loci were associated with CeAD susceptibility, and annotated functional genes had roles in 20 important GO terms clustered into biological process and molecular function. The PRS derived from risk variants was associated with CeAD incidence after adjusting for age and sex both in training test and validation.

**Supplementary Information:**

The online version contains supplementary material available at 10.1186/s12883-024-03759-0.

## Introduction

The incidence of stroke in young patients has increased considerably over the past few decades [1]. Although the incidence of cerebral artery dissection (CeAD) is estimated to be about 2.6-3 per 100,000 inhabitants per year, CeAD is a major cause of stroke in young adults [2]. Compared with older adults, young adults have more ischemic infarcts from CeAD - up to 6.8% [2]. Patients can present with various clinical symptoms, some of which are benign, but most patients have disabling symptoms, especially in those with subarachnoid haemorrhage and brainstem involvement [3]. Risk factors for CeAD include inherited connective tissue diseases, hyperhomocysteinemia, migraine, recent history of infection, and neck massage [4]. However, those risk factors often insufficient to explain the pathology of CeAD. Due to the low incidence and challenge in diagnoses, there are few study cohorts of CeAD worldwide.

Arterial dissection is separation of the arterial walls. It can occur in all large and medium sized arteries. Recently, studies have shown genetic predisposition of abdominal and thoraic aortic aneurysms and dissections (AAD). There are several genes that have been identified as strong causative risk for AAD through family studies [5, 6]. Yet the most of remaining AAD cases involved common genetic variants affecting many genes. In a European cohort of CeAD, rare genetic imbalance was associated with high risk of CeAD and functional outcome after ischemic stroke [7, 8]. Furthermore, this cohort study revealed that a common variation in *PHACTR1* was involved in the CeAD [9]. These results indicate that CeAD have complex origins, while the genetic predisposition of CeAD in Asian populations have not been reported yet.

To our knowledge, this is the first study for CeAD in Chinese population. In the current study, we explored a GWAS analysis of 210 CeAD patients and 280 control individuals and identified ten previously unpublished genome-wide significant loci located in ten genes. We further constructed polygenic risk scores (PRS) in our cases and constructed predictive model for CeAD by using age, sex and PRS as predictors in verification.

## Methods

### Study participants

We enrolled 210 CeAD patients and 280 control individuals from June 2017 to September 2022 in Department of Neurology, Huashan Hospital, Fudan University. The CeAD patients were selected from the Chinese Cervicocephalic artery dissection study (CCADS) [10]. The control group was from the Health Screening cohort in Huashan Hospital in the same period. This cohort was aimed to evaluate the genomic and epidemiological risk of cognitive impairment. The age and the gender of subjects were matched to the cases group.

### Genotype calling and quality control

Genotyping data of patients were derived from Exome sequencing in DNA samples relying on AmCareSeq-2000 System (Jiajian Biological Engineering Technology Co., LTD, Guangzhou) at recruitment, and genotyping data of controls were derived from GWAS chipInfinium Asian Screening Array Kit relying on Illumina BEADLAB System at recruitment. For each case, NovaSeq WES reads are mapped with BWA MEM (version 0.7.17) to the hg19 reference genome [11]. Small variants are identified with Samtools and reported as per-sample gVCFs in GATK (version 4.2.2.0) [12]. These gVCFs are aggregated with GATK CombineGVCFs into a joint-genotyped, multi-sample project-level VCF (pVCF). Then all cases were compiled to one pVCF by GATK HaplotypeCaller tool for downstream analysis. Single nucleotide variant (SNV) genotypes with read depth (DP) less than 20 are changed to no-call genotypes using Bcftools (version 1.14) [12]. After the application of the DP genotype filter, a variant-level allele-balance filter is applied, retaining only variants that meet either of the following criteria [13]: (1) at least one homozygous variant carrier; or (2) at least one heterozygous variant carrier with an allele balance (AB) between the cutoff values (0.15 ≤ AB ≤ 0.85). We further excluded single nucleotide polymorphisms (SNPs) with minor allele frequency (MAF) < 10%, genotype missingness > 5% or Hardy–Weinberg equilibrium test *P* < 5 × 10^− 5^. The pVCF was converted to Plink file set using Plink (version 1.9) [14]. Then genotype data of patients and controls were combined with only common variants reserved. These genotyping data were imputed with Impute (version 2) software by using 1000 Genomes Project phase 3 panels as reference [15]. We applied filters to achieve high quality variants with following criteria [16]: (1) INFO score (information metric) ≥ 0.7; (2) call rate ≥ 95%; (3) minor allele frequency (MAF) ≥ 10%; (4) Hardy-Weinberg equilibrium (HWE) ≥ 5·× 10^− 5^. The above filtering steps were performed step by step in Plink. Variants failing to meet these criteria will be filtered in subsequent analyses. We further excluded variants in MHC region (chr6 25-35 Mb) due to extensive linkage disequilibrium (LD). The final set of data contained a total of 821,475 variants.

### Population stratification

We compared the data set with the 1000 Genomes Project to infer the genetic ancestry of the population in this study according to the principal components (PCs) in 1000 Genomes Project phase 3 [17]. Based on the race of participants in the 1000 Genomes Project we used random forest classification to map the sample population to the most similar race. The results showed that all samples (cases and controls group) are clustered in the East Asian area (Supplementary Fig. S1). It can be concluded that participants in our analysis were derived from the East Asian ethnicity.

In the present study, we collected a total of 210 patients and 280 controls in two batches. The first batch included 140 patients and 210 controls, and the second batch included 70 patients and 70 controls. Participants in first batch were enrolled from 2019 to 2021 while the second batch were enrolled from 2021 to 2022 under the same diagnostic criteria. Data of the first batch were used in genome-wide association analysis (GWAS), while the second batch was used as external validation. Firth logistic regression test was used to test the association of SNPs with phenotype. Age, sex, genotyping array and first 10 principal components were adjusted for population heterogeneity in the multi-variable regressions.

### Annotation and pathway analysis

Independent significant SNPs were extracted when their P-values reach genome-wide significant threshold (*P* ≤ 5.0 × 10^− 8^) and in low LD (r^2^ < 0.1) with other SNPs within a 500-Kb window. Lead SNPs were identified as a subset of the independent significant SNPs with the lowest P-values and were in LD with each other at r^2^ < 0.01 within a 1-Mb window. The lead SNPs were mapped to nearest protein-coding genes within a 1-Mb window using locuszoom tool. To explore the function of significant genes, we annotated all independent SNPs with P value ≤ 5.0 × 10^− 5^ using snpEff and performed pathway analysis using R (version 4.2.2) package ‘clusterProfiler’. Kyoto Encyclopedia of Genes and Genomes (KEGG) pathway and Gene Ontology (GO) enrichment analyses were performed to identify the significant pathways. *P* < 0.05 was set as the cutoff criterion for significant enrichment.

### Predictive model and validation

We selected independent SNPs (r^2^ < 0.01 within a 1-Mb window) associated with CeAD based on GWAS results at P value threshold 5 × 10^− 5^ to construct polygenic risk score (PRS) in training dataset.

According to the previous study, PRS was referred to as genomic risk score, which was a method to predict genetic predisposition for disease of an individual. The calculation of PRS was the sums of the estimated effect sizes (β) of m SNPs, based on the estimated SNP effect sizes (β) obtained from GWAS summary statistics. (“effect sizes” refer to the measure of the strength of the association between a genetic variant and a particular trait or condition. The effect size is often represented by β. “m” is the count of genetic variants included in the PRS model.) The formula shows as below. X_ij_ means the genotype of i^th^ individual and j^th^ SNP [18, 19].

m

PRS_i_ = ∑x_ij_β_j_

j=1

In this study, PRS was calculated using PRSice-2 (version 2.3.5) as the sum of risk presented at each locus and weighted by the odds ratio for that locus for each participant. Next, PRS was evaluated for whether it could help identifying individuals at risk of developing CeAD in the validation dataset. Further, we constructed prediction models using logistic regression analysis with risk factors in the training set. Three models containing different predictors were constructed and the internal validation of the models was evaluated by using a ten-fold cross validation approach. Model 1 used PRS as predictor. Model 2 included age and sex as predictors. Model 3 involved age, sex and PRS together. Further, we compared three models’ performance in the validation set.

## Results

### Genome-wide association analysis of CeAD

**Fig. 1:**
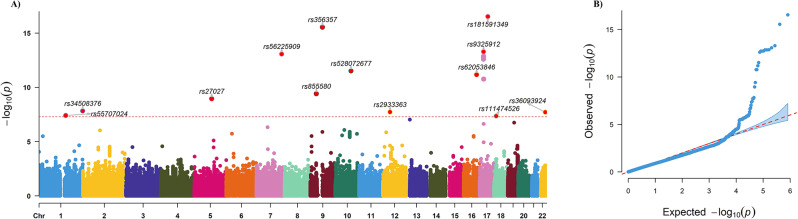
Result of Genome-wide association study on CeAD. **A**: Manhattan plot of susceptibility locus highlighting in CeAD GWAS. **B**: Q-Q plot for CeAD GWAS

A total of 490 participants were involved in our study, including 210 patients and 280 controls. The patients (mean age 41.6 ± 11.4 years) were younger than the controls (mean age 53.3 ± 7.6 years). We performed GWAS to identify genetic variants associated with CeAD using 140 patients and 210 controls in the training dataset. Thirteen lead SNPs were found to be associated with CeAD at a genome-wide significance level of *P* < 5 × 10^− 8^ (Fig. 1A; Table 1). Genomic inflation factor (λ_GC_) was estimated as 1.09, so P value was adjusted with λ_GC_ to diminish the impact of systematic inflation (Fig. 1B). Then, independent significant SNPs were annotated with functional genes residing in the location or within the upstream and downstream ± 500Kb regions (Table 1). The most significant SNP rs181591349 T/A (OR = 0.09, 95%CI 0.06–0.15) was at locus 17q21.32. We annotated lead SNPs with functional genes residing in the location or within the upstream and downstream ± 500Kb regions. Three SNPs (rs356357, rs528072677, and rs9325912) were excluded in the following analysis due to locating outside of protein-coding region. We also explored the association between lead SNPs and the severity of CeAD in patients, however, no significant association was found.

**Table 1:** Summary of lead SNPs associated with CeAD

Lead SNP	Gene	CHR	Position	A1	A0	OR	95% CI	*P*	MAF
rs55707024	FLG	1	152,276,282	G	C	0.23	0.15 ~ 0.37	3.91E-08	0.25
rs34508376	OR2L13	1	248,084,909	G	T	0.22	0.14 ~ 0.34	1.49E-08	0.24
rs27027	RGMB	5	97,912,527	C	G	6.85	4.00 ~ 11.7	1.12E-09	0.13
rs56225909	EPHB6	7	142,471,344	A	G	6.39	4.18 ~ 9.75	8.49E-14	0.43
rs855580	PRSS3	9	33,796,912	A	G	7.39	4.29 ~ 12.7	3.91E-10	0.13
rs356357	-	9	68,358,224	T	C	0.12	0.07 ~ 0.18	2.83E-16	0.42
rs528072677	-	10	88,994,492	T	C	7.99	4.82 ~ 13.3	3.07E-12	0.16
rs2933363	LRRK2	12	40,880,587	A	T	3.01	2.16 ~ 4.21	1.88E-08	0.4
rs62053846	PKD1L3	16	72,012,404	A	G	4.39	3.04 ~ 6.34	6.71E-12	0.32
rs9325912	-	17	21,535,937	T	C	0.16	0.11 ~ 0.25	5.15E-14	0.48
rs181591349	KPNB1	17	45,623,441	T	A	0.09	0.06 ~ 0.15	2.94E-17	0.44
rs111474526	GNAL	18	11,644,364	A	G	6.15	3.50 ~ 10.8	4.42E-08	0.11
rs36093924	CYP2D6	22	42,538,399	T	C	0.24	0.16 ~ 0.37	1.95E-08	0.26

### Functional annotation and enrichment analysis

**Fig. 2:**
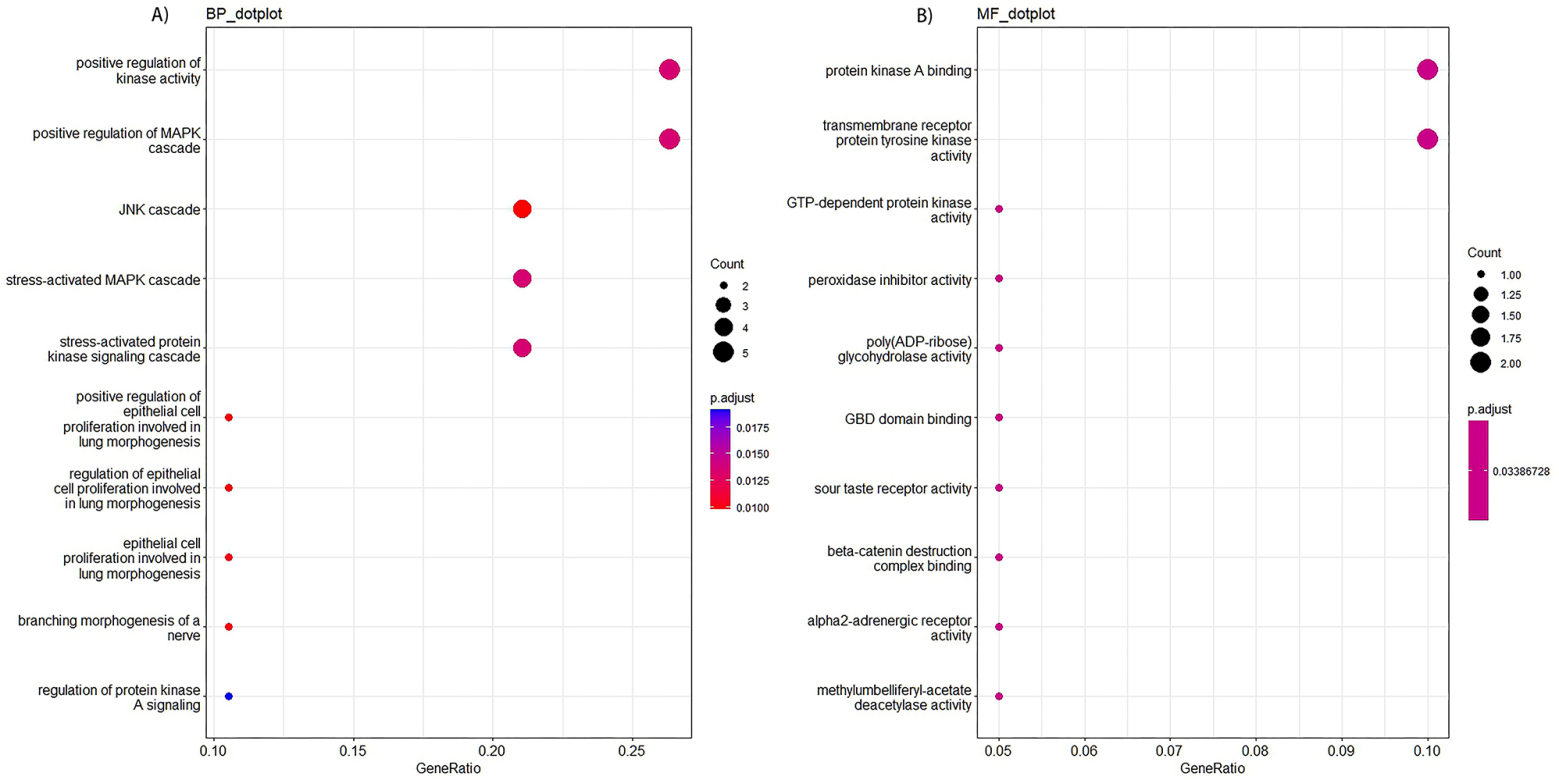
GO enrichment analysis results of differential genes associated with CeAD in biological process (**A**) and molecular function (**B**)

To explore the association between differential genes and CeAD, we included all independent SNPs with P value < 5 × 10^− 7^ and annotated them to nearby function genes within 1-Mb window. A total of 28 independent SNPs located in 20 protein-coding genes were included in enrichment analysis. We found that significant pathways were associated with biological process (BP) and molecular function (MF) in GO enrichment analysis (Fig. 2). Top 3 pathways in our gene set were GO:0033674, GO:0043410, and GO:0007254 for biological process, and GO:0051018, GO:0004714, and GO:0034211 for molecular function, respectively (Table 2). No significant cluster (*P* < 0.05) was found in KEGG pathway analysis.

**Table 2:** Summary of enrichment pathways associated with CeAD

ID	Description	Gene Ratio	*P* Value	*P* Adjust	Q Value	Gene ID
**BP**						
GO:0033674	positive regulation of kinase activity	5/20	8.83E-05	0.0135	0.0079	CDC42/ADRA2B/EPHB6/FGFR2/LRRK2
GO:0043410	positive regulation of MAPK cascade	5/20	0.0001	0.0135	0.0079	CDC42/ADRA2B/PJA2/FGFR2/LRRK2
GO:0007254	JNK cascade	4/20	2.52E-05	0.0098	0.0057	CDC42/PJA2/LRRK2/NPHS1
GO:0051403	stress-activated MAPK cascade	4/20	9.35E-05	0.0135	0.0079	CDC42/PJA2/LRRK2/NPHS1
GO:0031098	stress-activated protein kinase signaling cascade	4/20	0.0001	0.0135	0.0079	CDC42/PJA2/LRRK2/NPHS1
GO:0060501	positive regulation of epithelial cell proliferation involved in lung morphogenesis	2/20	9.66E-06	0.0098	0.0057	CDC42/FGFR2
GO:2,000,794	regulation of epithelial cell proliferation involved in lung morphogenesis	2/20	2.03E-05	0.0098	0.0057	CDC42/FGFR2
GO:0060502	epithelial cell proliferation involved in lung morphogenesis	2/20	3.47E-05	0.0101	0.0059	CDC42/FGFR2
GO:0048755	branching morphogenesis of a nerve	2/20	4.33E-05	0.0101	0.0059	FGFR2/LRRK2
GO:0010738	regulation of protein kinase A signaling	2/20	0.0002	0.0191	0.0111	PJA2/LRRK2
**MF**						
GO:0051018	protein kinase A binding	2/20	0.0013	0.0339	0.0173	PJA2/LRRK2
GO:0004714	transmembrane receptor protein tyrosine kinase activity	2/20	0.0019	0.0339	0.0173	EPHB6/FGFR2
GO:0034211	GTP-dependent protein kinase activity	1/20	0.0011	0.0339	0.0173	LRRK2
GO:0036479	peroxidase inhibitor activity	1/20	0.0011	0.0339	0.0173	LRRK2
GO:0004649	poly (ADP-ribose) glycohydrolase activity	1/20	0.0022	0.0339	0.0173	PARG
GO:0032427	GBD domain binding	1/20	0.0022	0.0339	0.0173	CDC42
GO:0033040	sour taste receptor activity	1/20	0.0022	0.0339	0.0173	PKD1L3
GO:1,904,713	beta-catenin destruction complex binding	1/20	0.0022	0.0339	0.0173	LRRK2
GO:0004938	alpha2-adrenergic receptor activity	1/20	0.0032	0.0339	0.0173	ADRA2B
GO:0047374	methylumbelliferyl-acetate deacetylase activity	1/20	0.0032	0.0339	0.0173	CES1

### Predictive model and validation

**Fig. 3:**
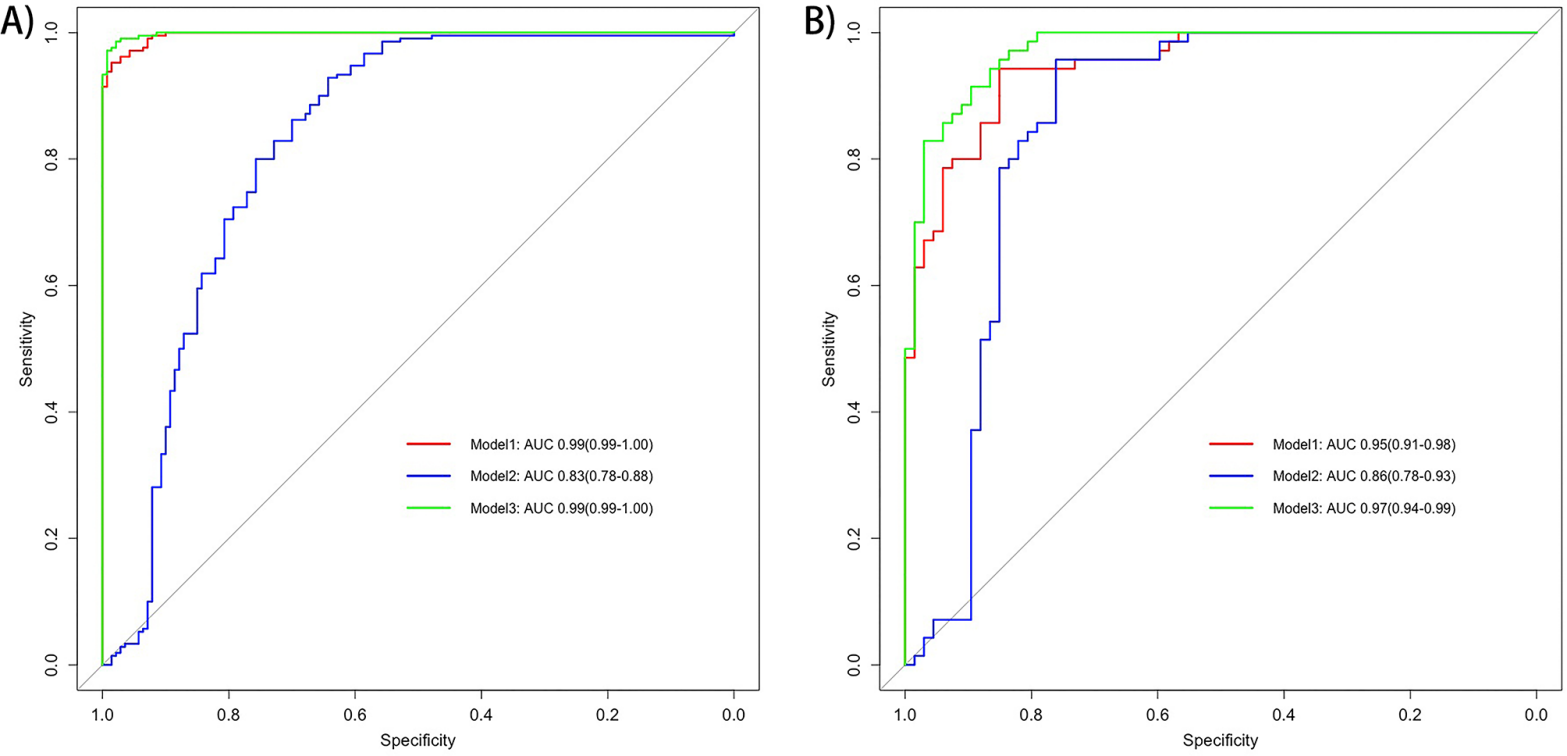
The receiving operator characteristic curve of three different models in training test (**A**) and validation (**B**) set

We set P value 5 × 10^− 5^ as threshold to construct PRS including 32 independent SNPs. Then we constructed predictive model for CeAD by using age, sex and PRS as predictors in the training set. The predictive ability of the model was estimated in validation set using area under the receiving operator characteristic curve (AUC). All three models could accurately predict the occurrence of CeAD both in training test and additional validation (Fig. 3). The AUC of Model 1 including only PRS reached 99% and 95% in training test and additional validation, respectively. The AUC of Model 2, including age and sex, reached 83% and 86% in training test and additional validation, respectively. The AUC of Model 3 including age, sex, and PRS together reached 97% (95%CI: 94 − 99%) in validation dataset.

## Discussion

To our knowledge, this is the first study to investigate the genetic predisposition of CeAD in Chinese population. We identified ten leadind SNPs located in ten protein-coding genes for CeAD. Then, we applied a PRS by capturing all independent GWAS SNPs and demonstrated a significant association of the PRS with CeAD incidence after adjusting for age and sex both in training test and extra validation.

The present results showed that CeAD heritability is polygenic and yielded the following two findings. First, patients with CeAD were more likely to carry the variations in the genes associated with protein kinase pathways. Among them, rs34508376 (OR2L13) was a suggestive role in CeAD pathophysiology which was in line with the previous observations in aortic aneurysms [20]. The other nine genes were first-time associations in CeAD cases. Second, the PRS derived from GWAS risk variants predicted occurrence of CeAD with high stability and consistency.

It is believed that CeAD is a complex disease due to various factors [21]. According to previous studies, history of migraine, mechanical trauma and preceding infection are commonly reported in CeAD [4, 22]. However, these factors were absent in more than half of the patients [23]. Some inherited connective tissue diseases are also associated with CeAD, such as Fibromuscular dysplasia (FMD), Ehlers-Danlos syndrome (EDS) and Marfan syndrome (MFS) [24], suggesting that genetic predisposition contributed to the occurrence of CeAD. However, these monogenic disorders cannot explain the genetic involvement in the remaining sporadic cases. Thus, large-scale genetic sequencing analyses are needed to explore more general genetic variants in CeAD patients. Through GWAS analysis of enrolled 140 cases and 210 controls in the training dataset, we identified 13 leading SNPs to be associated with CeAD at a genome-wide significance level of *P* < 5 × 10^− 8^. Among them, 10 SNPs were annotated on functional genes. These ten functional genes have known roles in 20 important GO terms clustered into BP and MF. Top 3 terms in our gene set were associated with protein kinase pathways. It was in line with the previous studies [25]. Other studies have shown that the main pathophysiologic features of arterial dissection are the impairment of vessel wall, especially the disruption of medial layer. Thus, it was previously thought that genes which encoded proteins involved in the structure or function of the vascular smooth muscle cells (VSMC) elastin-contractile unit were altered to cause aortic aneurysms and dissection [26]. Recent reports indicated that macrophage metabolic reprogramming and hyper-eosinophilic inflammation are involved in the aortic dissection. To be specific, macrophages in aortic dissection had higher levels of several glycolytic intermediates and tricarboxylic acid cycle (TAC) intermediates than control, which leaded to the secretion of inflammatory factors damaging the vessel wall [27]. In addition, the accumulation of eosinophils in the arterial wall released cytotoxic products and induced inflammatory response [28, 29]. These results were in accord with the risk genes in our genetic analysis that the arterial dissection is a complex disease involving many biological processes besides the impairment of VSMC unit.

The latest study did find evidence for olfactory receptor 2L13 (OR2L13) in growth of aortic aneurysms (AAA) [20]. OR2L13 regulates the platelet activation in AAA. Platelets from patients with AAA and murine models of AAA demonstrated increased OR2L13 expression. Due to the same pathogenesis of splitting up of the arterial wall, the histological hallmark of AAA and CeAD are similar. In addition to OR2L13, several other genes in our study were also linked to vascular disease. EPHB6 encodes the receptor tyrosine kinase that was proved to regulate the vascular smooth muscle cell contraction and endothelial cell, and contribute to the development of atherosclerosis [30, 31]. Although LRRK2 is a gene associated with Parkinson’s disease, it has also been shown to regulate the function of vascular endothelial cells and promote inflammation in endothelial cells [32]. Thus, with the identified genes in our study, more research is needed to uncover the pathogenesis of CeAD.

Due to the limited sample size, we applied a series of strict filters in the above GWAS analysis to ensure the reliability of the results. However, strict filter criteria will cause some rare variants to be excluded from the analysis. Some rare genetic variants were identified to be responsible for the spontaneous coronary artery dissection [33]. Therefore, it is important to use new methods, such as gene-level collapsing analysis [34, 35], to gain insight into the genetic architecture of CeAD in the future.

Polygenic risk scores (PRS) aggregate many genetic variants across the human genome into a single score and have predictive value for multiple common diseases [18]. In recent years, genome-wide association studies (GWASs) have revealed numerous susceptible genes and loci for CeAD, indicating a more efficient predication role of PRS in CeAD [36]. However, all published genomics studies were conducted in European-ancestry populations, with few studies in other populations. We set P value 5 × 10^− 5^ as threshold to construct PRS including 32 independent SNPs in our cohort and constructed predictive model for CeAD by using age, sex and PRS as predictors both in internal and external verification. The predictive ability of PRS model for CeAD was stronger than age and sex. These results provide evidence that CeAD is a disease with a genetic background. Analyses that include these risk SNPs will be effective in identifying new associations with CeAD.

However, several limitations of the present study merit consideration. First, although our research had the largest sample size so far in China, the relatively small number of participants may lead to weak statistical power for evaluating the relationships. Second, some clinical risk factors are not included in our study, and the genetic data were from different platform. However, we have carried out more stringent quality control and manual check. Longitudinal and multicenter studies with large sample sizes are needed to investigate the genetic predisposition of CeAD. Third, the study focuses on the Chinese population, which is valuable, but also means that the findings might not be generalizable to other ethnic groups, given that most published genomic studies have been conducted in European-ancestry populations. However, we supplemented the results on the Chinese population so that we can compare the findings from different populations to reveal potential population-specific genetic variations and contribute to a more comprehensive understanding of CeAD susceptibility across ethnicities.

### Electronic supplementary material

Below is the link to the electronic supplementary material.

Supplementary Material 1

## Data Availability

The datasets generated in the current study are available in the figshare, DOI: 10.6084/m9.figshare.25424824.
