# Supplementary material for: GWAS-based polygenic risk scoring for predicting cerebral artery dissection in the Chinese population
Source: BMC Neurol. 2024 Jul 25;24:258. doi: 10.1186/s12883-024-03759-0 (PMC11271197; doi:10.1186/s12883-024-03759-0)
Supplement: Supplementary file 1 — Supplementary Material 1 [file 12883_2024_3759_MOESM1_ESM.docx]

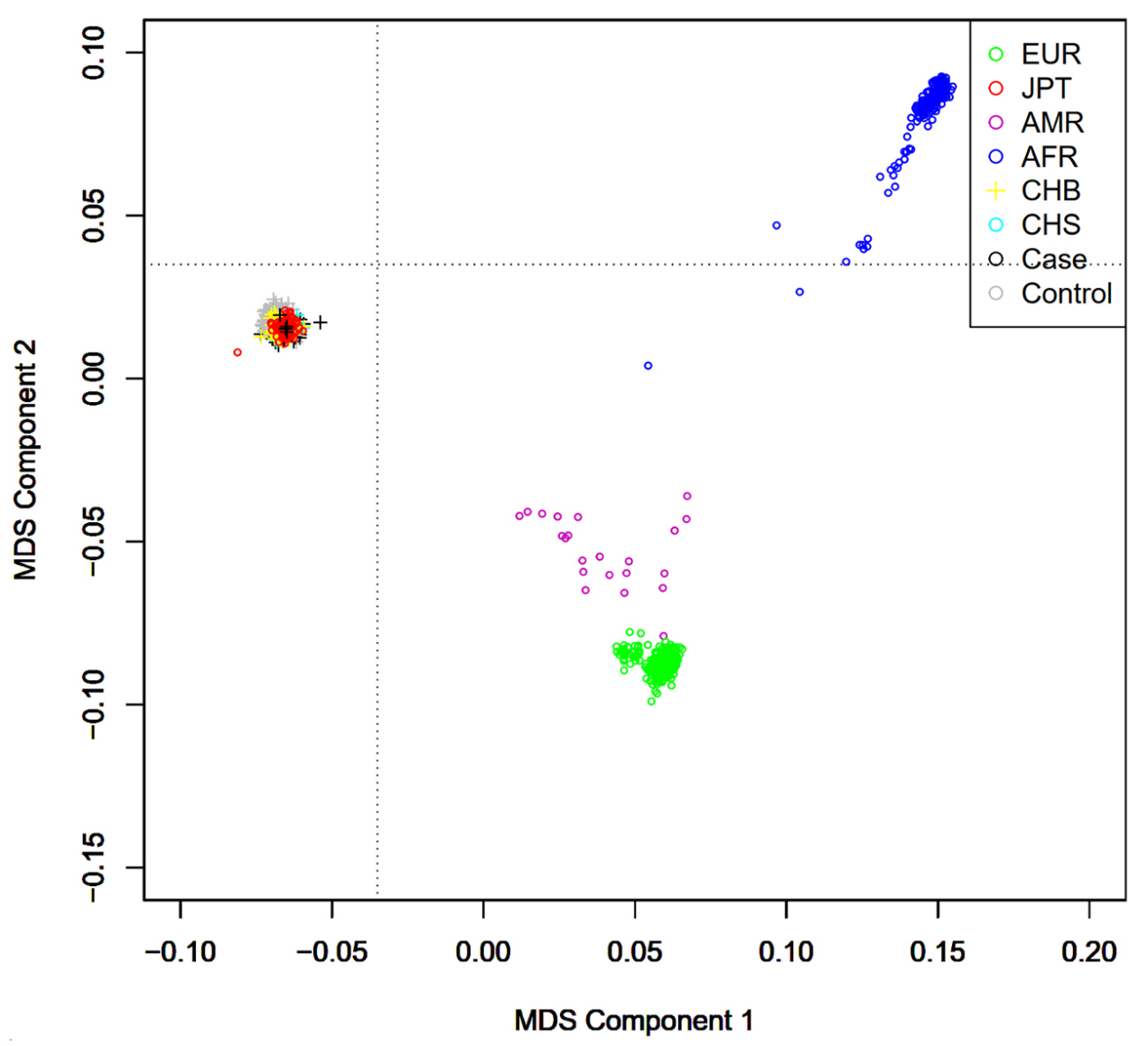


**Supplementary figure 1: Population stratification of individuals in our study**

**Genetic association analyses for CeAD**
